# Book Notices and Reviews

**Published:** 1864-02

**Authors:** 


### BOOK NOTICES AND REVIEWS.

*A Report on Hospital Gangrene, Erysipelas and Pyæmia*—As Observed in the Department of the Ohio and the Cumberland. With cases appended. By M. GOLDSMITH, Surgeon U. S. V. Louisville: Bradley & Gilbert, 1863. pp. 95.

This work is a valuable contribution to science, by a Surgeon of Volunteers. Its object is to show the value of Bromine in the treatment of Hospital Gangrene and Erysipelas. In this it is singularly successful.

The author was led to try this substance from the researches of Dr. Brainard on the effects of Iodine in snake bite and other poisonous wounds, but does not seem to be aware of his use of this remedy by infiltration in Erysipelas by injection into unhealthy suppurating cavities, etc., nor of the experiments of Reynoso on Bromine as an antidote to the woorara.

But to Dr. Goldsmith belongs the merit of using it in Hospital Gangrene and Erysipelas, whether in these diseases it is superior to Iodine further experience must determine. It is never volatile, and acts more energetically on the tissues, and cauterizes when injected into them.

The following are Dr. Goldsmith's directions for the use of Bromine:

1st. Give Chloroform if required.

2d. Remove all the sloughs with the scissors, so as to apply the pure Bromine upon the living tissues.

3d. It should be repeated if fetor remains.

The author advises a solution of his favorite remedy to be left standing in the wards to prevent infection.

The table on the following page gives the results of the treatment:

### CONDENSED TABULAR STATEMENT OF CASES OF HOSPITAL GANGRENE.

| | Whole number. | Recovered. | Died. | Amputations. | Average duration of treatment. | Percentage of deaths. |
|---|---|---|---|---|---|---|
| Treated with Bromine, in any way, - - - | 152 | 148 | 4 | 0 | 5 days and 14 hours | 2.65 (Bromine cases) |
| Treated with Bromine, pure, exclusively, - - | 27 | 25 | 2 | 0 | 2 " " $23\frac{1}{2}$ " | |
| Treated with Bromine, in solution, exclusively, - | 86 | 84 | 2 | 0 | 6 " " $11\frac{1}{3}$ " | |
| Treated with Bromine, pure, after the solution failed, | 8 | 8 | 0 | 0 | 12 " " 18 " | |
| Treated with Bromine, after Nitric Acid had failed, | 23 | 22 | 0 | 1 | 3 " " $16\frac{1}{3}$ " | |
| Treated with Bromine, after other remedies failed, - | 8 | 8 | 0 | 0 | 3 " " 4 " | |
| Treated with Nitric Acid exclusively, - - | 13 | 5 | 8 | 0 | 3 " " 14 2-5 " | 61.54 } 50 |
| Treated with other remedies exclusively, - - | 13 | 7 | 5 | 1 | 7 " " 13 5-7 " | 38.47 } |
| Treated with other remedies after Bromine had failed, | 4 | 4 | 0 | 0 | | |

This table, it will be seen, shows a result which the author does not seem to have noticed, viz: that the average results of cases treated by the solution are more favorable than those treated by pure Bromine, being 1 death in 32 of the former, and 1 in 13½ in the latter.

The results, however, are sufficiently favorable in either way to excite the admiration of any one at all acquainted with the history of Hospital Gangrene, so much so as to lead to the question whether it is the same disease.

Dr. McLeod states that the patients in his wards in the Crimea never lived over sixteen hours after the attack. The French removed their wounded early to the Bosphorous, and sixty bodies were thrown overboard from one transport ship during a passage of thirty-eight hours. The disease in the French hospitals "attacked not only open wounds, but cicatrices, and nearly every stump."

Delpech describes it as being fatal in a few hours, while Dr. Goldsmith gives the average duration of treatment, by all methods, as from three to twelve days.

We must, therefore, conclude that the disease, if it has appeared at all in our military hospitals, from which emanate these reports, has only done so in its mildest form, and in a way so closely resembling Erysipelas as scarcely to be distinguished from it. It may be added that in none of our armies has the mortality compared with that of European troops making campaigns in warm climates, a fact which speaks well for the medical department of the service, and may be due also, in some degree, to the agency of the Sanitary Commission, in furnishing supplies of fresh vegetables and other useful articles.

The following are the author's views of the method of employing his medicine in Erysipelas:

"In treating cases of Erysipelas with Bromine, the following rules are commonly followed:

1st. The face, for example, being the part affected, the parts are to be washed in soap and water, so as to remove all sebaceous matter, and then sponged in clean water.

2d. A mask of patent lint is to be prepared, large enough to cover and extend three inches beyond the erysipelatous area, and in this a ⊥ shaped incision is to be made for the accommodation of the mouth and nose.

3d. Another piece of lint, the exact counterpart of the first.

4th. A piece of oiled silk, large enough to cover the face and head, with a like ⊥ incision.

5th. Two pieces of lint large enough to cover the eye-lids are to be smeared with simple cerate and placed over the eyes.

6th. The first mask of lint is to be placed over the face.

7th. The second mask is to be wetted in the following solution: ℞—Bromine, ʒj; Bromide of Potassium, gr. xxx; Water, ℥x.—M.—and placed over the first mask.

8th. The oiled silk is now to be quickly placed over the face and secured by a roller; the object being to cause the vapor of the Bromine to come in a dry state in contact with the skin. Some surgeons prefer the use of the solution directly upon the skin.

9th. The application should be renewed once in four hours until the disease subsides.

Sometimes the above formula proves irritating. In that case the strength of the solution must be reduced by the addition of water."

The results are most favorable, the deaths being, in cases treated with Bromine topically and with vapor in the wards, not more than about five per cent.

---

*Synopsis of Lectures on Materia Medica and Pharmacy,* delivered in the University of Pennsylvania, with Three Lectures on the *Modus Operandi* of Medicines. By Joseph Carson, M. D. Third edition, revised. 8vo., pp. 244. Philadelphia: Blanchard & Lea, 1863.

This book is an outline of the course of lectures delivered by the author in the University of Pennsylvania. The synopsis evinces that the course is well arranged and complete. To be valuable to the members of the class, the outline thus given, should be filled up by the student, as the lectures are given. The three lectures on the *modus operandi* of medicines which are appended, are most ingeniously and carefully drawn up, and, in the present state of physiological science, may be received as correct.

*The Nervous and Vascular Connection between the Mother and Fœtus in Utero.* By John O'Reilly, M. D., F. R. C. S., I. New York: Printed by Robert Craighead, 1864.

From the author. Shall be noticed in the next number.

---

*Journals Received.*—The American Journal of Medical Sciences, (quarterly); Medical and Surgical Reporter, (weekly); Pacific Medical and Surgical Journal; Medical News & Library; The Journal of Ophthalmology; American Medical Times, (weekly); Medical Examiner; Boston Medical and Surgical Journal, (weekly); Canada Lancet; London Lancet; Buffalo Medical and Surgical Journal; Dental Register; People's Dental Journal; Cincinnati Lancet and Observer; The American Exchange and Review; American Journal of Insanity; Braithwaite's Retrospect for January, 1864.

